# Temporal changes in the proportion of *Salmonella* outbreaks associated with 12 food commodity groups in the United States

**DOI:** 10.1017/S0950268822001042

**Published:** 2022-06-15

**Authors:** Michael S. Williams, Eric D. Ebel

**Affiliations:** United States Department of Agriculture, Risk Assessment and Analytics Staff, Office of Public Health Science, Food Safety Inspection Service, 2150 Centre Avenue, Building D, Fort Collins, Colorado 80526, USA

**Keywords:** Compositional data, salmonellosis, signal-to-noise ratio

## Abstract

Using data from 20 years of *Salmonella* foodborne outbreaks, this study investigates significant trends in the proportion of outbreaks associated with 12 broad commodity groups. Outbreak counts are demonstrated to have a stronger trend signal than outbreak illness counts. The number of outbreaks with an identified food vehicle increased significantly between 1998 and 2000. This was followed by a 10-year period when the number of outbreaks decreased. The number of outbreaks increased significantly between 2010 and 2014 and then remained unchanged for the remainder of the study period. During the period of 1998 through 2017, the proportion of outbreaks for three commodities groups, consisting of eggs, pork and seeded vegetables, changed significantly. No significant changes were observed in the remaining nine commodity groups. Simple approximations are derived to highlight the effect of dependencies between outbreak proportions and a consumption analysis for meat and poultry is used to enhance the limited interpretability of the changes in these proportions. Given commodity-specific approaches to verifying food safety and promoting pathogen reduction, regulatory agencies benefit from analyses that elucidate illness trends attributable to the products under their jurisdiction. Results from this trend analysis can be used to inform the development and assessment of new pathogen reduction programmes in the United States.

## Introduction

In the United States, the Department of Agriculture's (USDA) Food Safety and Inspection Service (FSIS) maintains jurisdiction and inspection over meat, poultry and egg products. The Department of Health and Human Services' Food and Drug Administration (FDA) is responsible for all other commodities. State and local governments also maintain some authority to regulate commodities within their more limited geographic jurisdictions. Across these different agencies, many programmes have been implemented to encourage pathogen reductions in some of these commodity groups. An example is the Pathogen Reduction; Hazard Analysis and Critical Control Point (PR;HACCP) regulation [1]. One of the goals of this regulation was to reduce pathogenic microorganisms on meat and poultry products, thereby reducing the incidence of foodborne illness attributed to consumption of these products.

While both FSIS and FDA collect samples of different food commodities and monitor for changes in the occurrence of pathogen-positive product samples, it is difficult to link changes in an individual commodity, or group of commodities, to overall changes in the total burden of illness. Analyses of changes in contamination rates for an individual commodity, without considering the possibility of concurrent changes to other commodities, have led to discrepancies in the degree to which changes in human illnesses have been linked to different commodities [2–4]. Estimating the change in an individual commodity's contribution to overall foodborne illness requires an assessment of changes within the context of foodborne illnesses for all commodities. This requires understanding the proportion of estimated illnesses for a specific pathogen attributed to specific commodities. These proportions are referred to as attribution fractions. In the United States, the Interagency Food Safety Analytics Collaboration (IFSAC), a collaboration amongst the Centers for Disease Control and Prevention (CDC), FDA and FSIS, works together to generate national estimates of the proportion of illness attributed to 17 broad commodity groups [5].

Multiple methods and data types exist to estimate pathogen-specific attribution fractions for various commodities [6–16], and many methods use national datasets of foodborne disease outbreaks to attribute observed illnesses to specific commodities implicated in pathogen-specific outbreaks. The advantage of using outbreak data is that the epidemiological evidence collected from outbreak investigations offers a direct linkage between a commodity and human illness. In contrast, methods such as the microbial subtyping approach [17] or methods based on characteristics derived from whole genome sequencing (WGS) [18] usually only provide evidence of an association between a pathogen's host species at some point prior to human illness without identifying the specific food vehicle. Despite the differences in methods and data, one commonality of all attribution methods is that they estimate a surrogate variable that is assumed to be representative of the true proportion of illnesses associated with a specific product–pathogen pair.

In the United States, outbreaks attributed to *Salmonella*-contaminated foods provide the most robust data source available for the attribution of illnesses to different commodities. This is due to the large number of outbreaks, relative to the other foodborne bacterial pathogens [19], the occurrence of *Salmonella* outbreaks across all 17 commodity groups [20, 21] and the general similarity between the characteristics of sporadic cases identified through laboratory surveillance and outbreak cases [22]. In recent years, FSIS has focused on *Salmonella* and has sought to utilise programmes and policies to promote the reduction of *Salmonella* and other pathogens in meat, poultry and egg products [23].

This study examines 20 years of data for *Salmonella*-attributed foodborne outbreaks to determine if significant trends for various commodities can be identified. Topics include variable selection and models for identifying significant trends. While the analysis for this study necessarily includes commodity groups that fall outside of FSIS’ jurisdiction, the presentation of results will focus primarily on meat and poultry products.

## Data description

The United States has a long history of surveillance for food and waterborne outbreaks, dating back to surveillance for milk-attributed outbreaks beginning in 1923. In 1998, state and local health departments began submitting foodborne outbreak reports directly into CDC's Electronic Foodborne Outbreak Reporting System (eFORS) [24]. In 2009, the surveillance system was expanded beyond foodborne outbreaks to capture data on waterborne outbreaks and outbreaks transmitted by contact with animals, people or the environment, and outbreaks with an unknown mode of transmission. At this time the reporting platform was renamed the National Outbreak Reporting System (NORS) [25]. Detailed descriptive statistics of the NORS, including temporal and spatial patterns, are available in earlier publications [26–28]. The publicly available data from NORS were extracted for the period 1998–2017 [29]. This database contains summary information for all bacterial foodborne outbreaks, some of which have a single identified food vehicle. Each of the implicated food vehicles has been grouped into one of 17 broad commodity groups [20, 21]. This dataset represents 2712 *Salmonella* foodborne disease outbreaks, of which 863 outbreaks have a single identified food vehicle. The total number of illnesses associated with all outbreaks was 72 412, and the total number of illnesses among food vehicle-identified outbreaks was 34 082.

An assessment of the outbreak data by commodity groups finds that many of the 17 groups consistently experience an average of less than one *Salmonella* outbreak per year. For example, the commodity group consisting of oil and/or sugar-related *Salmonella* outbreaks consists of a single outbreak during the 20-year period. Similarly, there were only two outbreaks associated with the commodity group of *game*. To address this issue, the 17 groups were further combined into 12 groups such that each group had close to a minimum of one outbreak in more than 10 of the 20 years. This was accomplished using the following approach: the group of *other meat/poultry* was combined with the *game* group; all aquatic protein groups were combined; the group of *other vegetables* was combined with *vegetable row crops*; the group of *oils and sugars* was eliminated. The resulting dataset is given in Table 1.

**Table 1.** Data and summary statistics of annual outbreaks using the 12 commodity classes

	Food categories												
Metric	Beef	Chicken	Dairy	Eggs	Fish and other seafood	Fruits–nuts	Other meat/poultry and game	Pork	Seeded vegetables	Sprouts	Turkey	Vegetable row crops and other vegetables	All
Mann–Kendall	0.370	0.819	0.267	0.001	0.090	0.066	0.772	0.003	0.019	0.517	0.547	0.283	0.229
Mean of ratios	0.09	0.19	0.05	0.18	0.04	0.10	0.02	0.11	0.06	0.05	0.08	0.04	NA
Mean	3.7	8.4	2.3	8	1.6	4.3	0.7	4.6	2.6	2	3.3	1.9	43.2
Variance	3.7	12.4	3	34.8	1.9	9.4	0.7	7.7	2.6	1.4	3.1	1.6	81.8
Outbreak counts													
1998	3	6	3	8	2	1	1	1	1	1	2	0	29
1999	2	17	1	24	0	7	1	0	0	4	3	1	60
2000	4	10	0	22	4	3	0	5	1	2	6	2	59
2001	4	9	3	9	1	6	0	5	0	3	1	1	42
2002	6	6	3	7	0	2	2	2	5	1	4	3	41
2003	9	8	1	12	1	4	1	3	1	2	2	1	45
2004	4	14	2	9	1	0	1	6	2	1	8	3	51
2005	5	7	3	12	1	1	0	1	3	1	4	2	40
2006	2	6	3	3	0	3	0	3	4	1	3	2	30
2007	5	5	4	5	1	1	0	6	3	3	2	3	38
2008	3	5	1	7	2	5	0	1	2	1	6	1	34
2009	4	3	0	7	2	2	1	4	3	4	1	4	35
2010	1	4	0	7	1	3	0	6	3	4	3	1	33
2011	2	7	1	1	0	8	0	10	3	3	4	0	39
2012	4	8	0	7	1	7	1	3	2	1	3	1	38
2013	5	10	5	3	2	8	3	8	5	1	3	0	53
2014	2	13	3	2	5	6	1	6	5	1	2	3	49
2015	1	10	5	5	4	1	0	8	4	1	4	4	47
2016	2	9	5	5	2	7	1	7	4	3	2	2	49
2017	5	11	3	5	2	11	0	7	1	1	2	3	51
Total	73	168	46	160	32	86	13	92	52	39	65	37	863

To aid in the interpretation of changes in the proportions of outbreaks it is helpful to understand changes in consumption patterns. The USDA Economic Research Service (ERS) Food Availability Data System (FADS) has provided estimates of annual per capita consumption for broad food commodity groups since 1909 [30]. The loss-adjusted food availability estimates serve as proxies for the total number of servings of each commodity at the national level. These estimates account for factors such as the differences in spoilage and waste for each commodity but do not account for the possibility of annual changes in serving size. This analysis will assume that serving size has remained constant. Given this study's primary focus on FSIS-regulated meat and poultry commodities, only the effect of changes in consumption for the beef, chicken, pork and turkey commodity groups will be considered. These estimates are derived from an annual census of the number of animals slaughtered, so the only sources of uncertainty are the adjustment factors applied to convert slaughter totals to servings. Annual beef consumption is derived from the sum of beef and veal consumption to be consistent with the hierarchy used to classify outbreaks [20].

## Methods

The analyses are divided into two parts. The first analysis is a variable selection process to determine the best variable for assessing changes in the proportion of outbreaks for each commodity. This analysis investigates the temporal patterns in the number of annual outbreaks and the number of annual illnesses associated with outbreaks. The most appropriate variable was then used for the assessment of trends.

The second set of analyses will determine if significant changes in the proportion of outbreaks attributed to any of the 12 commodity groups have occurred. The study will focus primarily on outcomes relevant to the FSIS-regulated commodities of beef, chicken, pork and turkey. Nevertheless, all commodity groups must be considered due to the correlated nature of proportions that share common measurements [31].

The final analysis is an investigation of the effects of changes in consumption patterns on the probability of illness associated with the meat and poultry commodities.

### Variable selection

A decision to use outbreak or illness counts in an estimation strategy depends on two factors, with the first being whether one of the variables is better suited to the modelling of trends across time. The second is to determine whether there are significant differences between the outbreak counts and illness counts that might lead to contradictory conclusions.

The estimation of temporal trends is dependent on the strength of the underlying temporal pattern compared to the degree of variability in the data, which can be defined by the signal-to-noise ratio given by
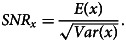
The variable with the larger signal-to-noise ratio will be better suited for the estimation of trends.

To investigate the *SNR* statistic for outbreak counts, assume the annual number of outbreaks can be modelled as *O*_*t*_ ~ Poisson(*λ*), where *t* is the indicator for year *t*. Using this model, the signal-to-noise ratio for outbreak counts is
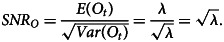


For each outbreak *j* in year *t*, let *S*_*tj*_ be the number of illnesses and assume a general discrete distribution with mean and variance terms defined by *S*_*tj*_ ~ (*μ*, *σ*^2^). Then the number of illnesses in year *t* is defined by the random sum 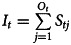. The properties of a variable that is a random sum are such that
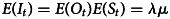
and
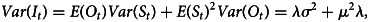
which yields
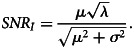


Given the signal-to-noise ratio values for outbreak *vs.* illness counts, we can determine under what conditions *SNR*_*I*_ ≥ *SNR*_*O*_. Investigating this inequality yields
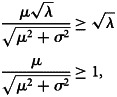
with the condition of equality satisfied only for *σ*^2^ = 0. It might be reasonable to use illness counts if *σ*^2^ were small relative to *μ*^2^, but a previous analysis of outbreak illness counts found that they are highly variable and remain heavily right-skewed even after applying a log transformation [32].

We conclude that significant trends are more likely to be observed using the outbreak counts variable *O*_*t*_ because it necessarily has a larger signal-to-noise ratio. The effect of the higher signal-to-noise ratio of *O*_*t*_ can be quantified by comparing the widths of the confidence intervals relative to the estimated trend model, described below, that was fitted to *O*_*t*_ and *I*_*t*_. For example, if 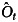 is the fitted value at time *t*, the average relative interval width statistic is
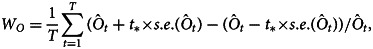
where 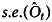 is the estimated standard error and 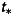 is the *t*-statistic derived from the fitted model described below.

The second step in the variable selection process is to determine what, if any, differences can be detected between the annual occurrence of outbreaks and illnesses for each commodity. To start, we assessed the correlation between the number of outbreaks and illnesses [33]. Next, an analysis of variance model is used to determine if significant differences exist in the number of illnesses per outbreaks for any of the commodity groups.

### Trends

The goal of this study is to determine if significant trends exist in the proportion of outbreaks associated with any of the commodities. If the total number of outbreaks or illnesses is constant across time, then changes in the annual number of outbreaks or illness for a commodity group, rather than the ratio of outbreaks in a commodity group relative to the total number of outbreaks, can be modelled using a single methodological approach.

The simplest test for possible trends is a Mann–Kendall test [34] applied to each of the commodity groups to assess if a significant monotonic trend was present in the data. To test for trends that are not necessarily monotonic, a general additive model was fitted to the data. For the outbreak counts, the model used for outbreaks is of the form
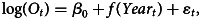
with *t* = 1998, 1999, …, 2016, 2017 and *f*(*Year*_*t*_) being a penalised B-spline regression function with a Poisson link function, as described in previous food safety applications [35–37]. The Bayesian information criterion is used to select the smoothing parameters of the best fitting model [38]. This model employs a visual interpretation for significant trends using a horizontal line test. A significant trend is indicated in any situation where a horizontal line, drawn across the graph, intersects both the lower and upper bounds of the interval. For this study, the lower and upper bounds are defined as the 2.5th and 97.5th percentiles. Therefore, a horizontal line that fails to intersect these boundaries results in a conclusion that we cannot reject the null hypothesis of no trend at an *α* = 0.05 level of significance. Estimation of this model's parameters was performed using the R software package Mixed GAM Computation Vehicle **(**mgcv) [39, 40]. This model was fitted to summarise the annual totals *O*_*t*_ and *I*_*t*_. The confidence intervals determine the average relative confidence interval width statistics (*W*_*O*_, *W*_*I*_).

Interpretation of trends in the proportion of outbreaks or illnesses associated with a specific commodity is not straightforward because a change in one commodity necessarily affects the proportions for all remaining commodities. The term *spurious correlation* was used to describe this phenomenon at the end of the 19th century [31]. This effect can be demonstrated by considering a simplified example. Suppose that outbreaks in year *t* consist of only two groups, with the groups being outbreaks associated with commodities derived from animals (*o*_*ta*_) and the other group being outbreaks from plant-derived foods (*o*_*tp*_). Furthermore, assume the number of animal-associated outbreaks is increasing linearly (*o*_*ta*_ = *α*_*a*_ + φ_*a*_*t*, with *α*_*a*_, φ_*a*_ > 0) with time, while the number of plant-associated outbreaks remains constant (*o*_*tp*_ = *α*_*p*_). The proportion of plant-associated outbreaks is
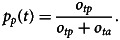


The effect of the increase in animal-associated outbreaks on the proportion of plant-associated outbreaks can be determined by considering the first derivative, given by1
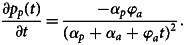


This example demonstrates that a significant change in the annual proportion of outbreaks associated with a specific commodity category is necessarily offset by changes in the opposite direction in the remaining categories. Furthermore, this relationship demonstrates why changes in the proportion of outbreaks for any single commodity cannot be considered in the absence of the remaining commodities.

The trend model uses the ratio of the number of outbreaks in year *t* attributed to food category*d*, denoted *o*_*t*,*d*_, *d* = 1, …*D*, with respect to the total number of outbreaks from the year, denoted *O*_*t*_. The term
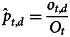
is the proportion of outbreaks in category *d* at time *t*. A similar model can be constructed for outbreak illnesses. The constraint that the *d* proportions add to one implies that they are not independent of one another (i.e. the proportions are negatively correlated because if the proportion for one category increases, this increase must be offset by decreases in other categories). As a result, these proportions cannot be analysed using statistical methods that are intended for unconstrained data [31, 41]. The solution employed is to use the additive log-ratio transformation technique used in compositional data analyses [42, 43], which reparametrises the outbreak count variable as
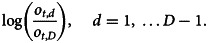


The data were fitted to a multivariate linear model of the form
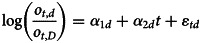
and tested for significance of the *α*_1*d*_ and *α*_2*d*_, where the vector of error terms is
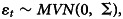
with Σ being the *D* − 1 × *D* − 1 covariance matrix. A quadratic model was also tested for each commodity, though none were significant. A similar model could be constructed using illness counts, but these results are not presented because the signal-to-noise results from above are still applicable.

In cases where no outbreaks occurred for commodity group *d* in year *t*, the fraction *o*_*td*_/*o*_*tD*_ was set to 1% of the fraction of outbreaks for that commodity. Parameter estimates are obtained using multivariate linear regression and the trend model for each category *d* is obtained by back-transforming the model predictions. Commodity group *D* is a reference group, and it consists of all outbreaks not being considered as part of the specific analysis. The trend for commodity group *D* is determined from the constraint that the sum of the proportions is equal to one. The results of the multivariate linear model are used to determine which commodities demonstrated significant changes in the proportion of outbreaks using a framework similar to one developed previously for assessing trends in antimicrobial resistance [42].

### Consumption analysis

The goal of the meat consumption analysis is to provide basic estimates of the change in the number of servings between 1998 and 2017 for the beef, chicken, pork and turkey commodity groups. The change is estimated by fitting a simple linear model of the form
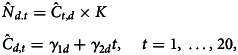
where 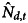 is the number of servings per year (or capita) for commodity group *d*, 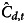 is the annual consumption (kg) estimate per capita for commodity group *d* and *K* is a constant that converts consumption data to servings per year (or capita). For example, if it is assumed a typical serving size of 100 g applies to all commodities, then *K* = 10 servings per kilogram consumed per capita per year.

The proportional change in annual number of servings is estimated as
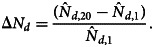


Assuming a constant serving size, an improved understanding of the factors affecting changes in the estimated proportion of outbreaks for each commodity can be derived by considering the relationship for any year *t*2
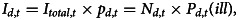
where *I*_*d*,*t*_ is the number of illnesses from the consumption of commodity *d*, *I*_*total*,*t*_ is the total number of foodborne illnesses from all commodities, *p*_*d*.*t*_ is the proportion of outbreaks from commodity *d* (assumed to be equivalent to the true attribution fraction of illness cases) and *P*_*d*.*t*_(*ill*) is the probability of illness per serving. The total number of domestically acquired illnesses per year can be estimated from the reported case rate per 100 000 [44] multiplied by the total population of the United States [45], adjusted for foreign travel and non-foodborne cases [46]. The *P*_*d*.*t*_(*ill*) term can be decomposed to describe multiple risk factors [47], such as dose concentration and difference in virulence between serotypes. Efforts undertaken by government or industry to reduce contamination would also be captured by changes in *P*_*d*.*t*_(*ill*).

By assuming serving size is constant across time and commodities, an empirical measure of the change in the probability of illness per serving for commodity group *d* is estimated as
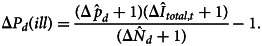


As described for 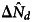, the other changes are measured based on the linear difference between 2017 and 1998. This estimate of the change in the probability of illness per serving is intuitive because its numerator estimates the change in annual illness counts (as the product of the attribution fraction and total illnesses) and its denominator estimates the change in total servings. Nevertheless, a linear model fitted to the case rates of salmonellosis from 1998 through 2017, based on culture-confirmed test results [44] has no significant slope term (*p* = 0.156).

## Results

### Comparison of outbreak and illness counts (variable selection)

**Fig. 1.**
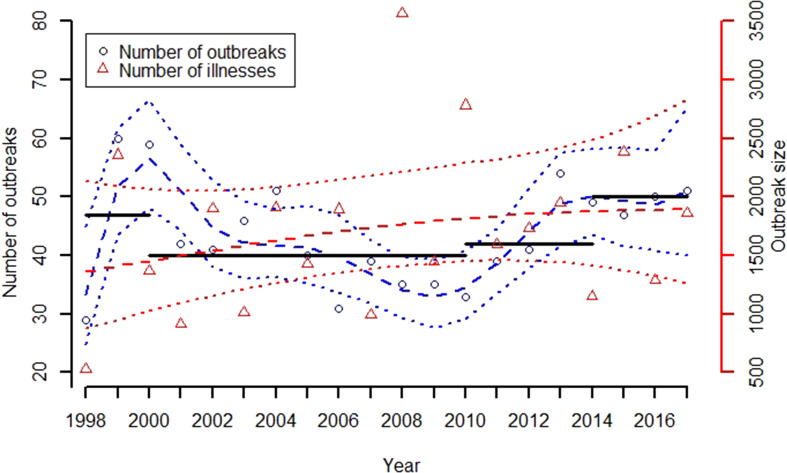
Penalised B-spline regression fits and confidence intervals for outbreak counts (blue) and illness counts from outbreaks (red). The horizontal solid lines are used to test for significant trends.

Figure 1 describes the fitted penalised B-spline models for both annual outbreak and outbreak illness counts. The outbreak counts demonstrate a rapid rise due to the doubling of outbreak counts between the first year (29 outbreaks), and the second and third years (60 and 59, respectively). This increase has been attributed previously to the 1998 introduction of an electronic reporting system for outbreaks [32]. This increase is followed by a roughly 10-year period (2001–2010) of statistically significant decline, where the average number of outbreaks drops to a low of roughly 35. The trend is reversed, beginning in the 2009–2010 timeframe, with a statistically significant increase through 2014. The annual number of outbreaks stabilises in the high 40s per year from 2014 through 2017.

The fitted penalised B-spline models for illness counts demonstrates a monotonic and nearly linear increasing trend in the number of illnesses; however, this increase is not statistically significant. A Cox and Stuart trend test [48] also fails to identify a significant trend (*p* = 0.37).

The effect of the additional variability associated with including the number of illnesses can be quantified by considering the relative width of the confidence intervals (e.g. the proportional difference between the ranges of the confidence interval relative to the estimated mean). Averaging across the 20 years, the average relative width of the confidence interval for the model based on the number of outbreaks is *W*_*O*_ = 0.34, whereas this value for illness counts is nearly double at *W*_*I*_ = 0.59. In addition, a Pearson's product moment correlation test failed to find a significant correlation between outbreaks and either illness counts (*ρ* = 0.01, *p* = 0.98) or log-transformed illness counts (*ρ* = 0.15, *p* = 0.55).

To further investigate the relationship between outbreak counts and illness counts, an analysis of variance model was used to assess if the number of illnesses per outbreak differed significantly by commodity. Of the 17 commodity groups in the dataset, prior to collapsing groups, only the seeded vegetables variable was significantly different (*p* = 0.0045). A likely cause of the significant difference is the number of large outbreaks associated with tomatoes, cucumbers and peppers. Removing the illnesses attributed to seeded vegetables and refitting the model indicates no significant difference across the remaining commodities groups (*p* = 0.64). Thus, these analyses suggest that the average annual number of cases of salmonellosis from outbreaks with an identified food source has remained constant at roughly 1700 illnesses per year, but that the power to detect a significant change in the number of illnesses is low in comparison to the number of outbreaks per year.

### Trends by commodity

After reducing the number of commodity groups from 17 to 12, only the group consisting of other meat-game has an average of less than one outbreak per year. Nearly 60% of all outbreaks occur in the groups of chicken, eggs, pork and seeded vegetables (Table 1). The Mann–Kendall test suggests significant monotonic trends in the annual number of outbreaks for the eggs, pork and seeded vegetables commodities (*p* = 0.001, 0.003, 0.019, respectively). Two of these commodity groups (i.e. eggs and pork) also have means and variances of their count data that differ substantially, indicating a departure from a simple Poisson process with a constant rate parameter.

**Fig. 2.**
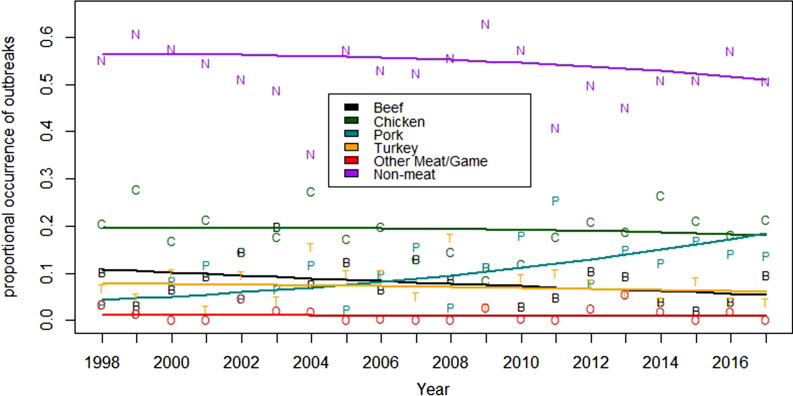
Compositional regression models for the five commodities groups and one group consisting of the remaining non-meat commodities in the United States. Pork is the only commodity group where the trend is statistically significant.

Outputs of the compositional trend model are used to assess changes in the proportion of outbreaks attributed to the 12 food commodity groups between 1998 and 2017. The compositional trend model for the five meat and poultry commodity groups is given in Figure 2. Amongst these commodity groups, only pork demonstrates a significant increasing trend (*p* = 0.006), with the trend in the proportion of outbreaks increasing from 0.04 to 0.18 between 1998 and 2017, respectively. While the trend model for beef decreases from roughly 0.10 to 0.05 during the study period, the decrease is not significant (*p* = 0.21). The proportion of outbreaks for the commodity groups of chicken, turkey and other meat/game is essentially unchanged during the 20-year study period. The trend in the proportion of outbreaks for all other outbreaks (i.e. the reference group *D*) is also included in Figure 2 to demonstrate that the proportion of outbreaks from non-meat commodities remained roughly constant at slightly more than 0.5.

**Fig. 3.**
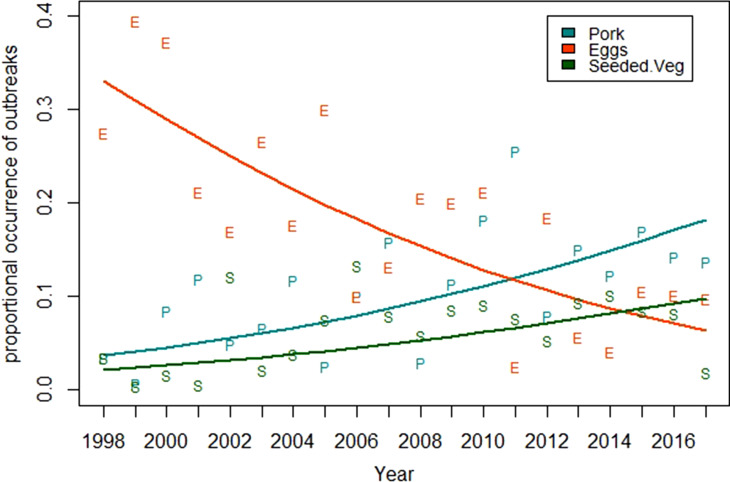
Trend models for the three commodities groups that demonstrated statistically significant changes (*p* = 0.005) in the proportion of outbreaks between 1998 and 2017.

As was the case with all meat and poultry commodities, other than pork, most of the remaining commodity groups exhibit no significant trends. Figure 3 shows the results of the application of the compositional data analysis approach to identify all commodity groups that demonstrate a significant trend. Only the commodity groups consisting of eggs, pork and seeded vegetables demonstrate significant overall changes (*p* = 0.005). This analysis finds that the commodity group of seeded vegetables increases from 0.02 to 0.1.

**Fig. 4.**
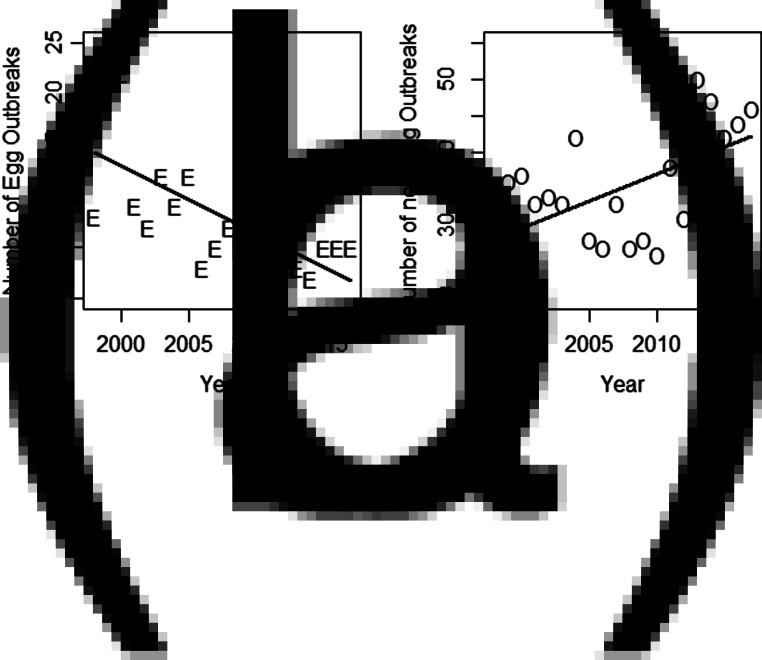
Changes in the number of egg-associated (a) and non-egg-associated outbreaks (b) during the study period. The substantial decrease in the number of egg-associated outbreaks inflates the proportion of outbreaks associated with all other commodity groups and highlights the difficulty of interpreting compositional data.

The proportion of egg-associated outbreaks demonstrated the largest reduction of any commodity group. The trend for this commodity monotonically and significantly decreases from 0.33 in 1998 to 0.06 in 2017 (Fig. 3). The effect of the reduction in this single commodity on the 11 remaining commodity groups can be estimated using equation (1), which shows that if there were no changes in the number of outbreaks for all other commodities, the estimated cumulative effect of the reduction in egg-associated outbreaks would be an increase in the proportion of outbreaks across the remaining commodities of 0.25. This estimate is conservative because the number of non-egg-associated outbreaks actually increases significantly by roughly 50% during the study period (Fig. 4). Applying simple linear models to describe the decrease in egg-associated outbreaks, fitted to the data in Figure 4, results in an estimated increase in the proportion of non-egg-associated outbreaks of 0.30. The consequence of this result is that the proportion of outbreaks for the non-egg-associated commodities is expected to increase as a result of the decrease in egg-associated outbreaks, particularly in the 1998–2008 timeframe when the annual decrease in egg-associated outbreaks was the greatest. This observation suggests a more nuanced interpretation of the results presented for the meat and poultry commodities (Fig. 2) because the commodities that demonstrated no change in their proportion of outbreaks (i.e. chicken, turkey and other meat/game) would be expected to increase somewhat as a consequence of the reduction in egg-associated outbreaks.

### Limits of the interpretability of compositional data

Further investigation of the meat and poultry commodities highlights the difficulty of generalizing the changes in the proportion of outbreaks to meaningful measures of risk for a commodity group. A recognised limitation of compositional data methods is that while changes in the proportions can be observed, it is not possible to determine why the change occurred and what factors may have contributed to the change [49]. Using the chicken commodity group as an example, not only does the proportion of outbreaks remains constant (Fig. 2), the best fitting linear model for the number of chicken-associated outbreaks consists of only the intercept term (i.e. *o*_*chicken*,*t*_ = 8.54), suggesting that the reduction in egg-associated outbreaks is offset by increases in commodities groups other than chicken (equation (1)), so that both the total number and proportion of chicken-associated outbreaks remain essentially constant.

A comparison of the results for the meat and poultry commodities also demonstrates that changes in the proportion of outbreaks, or lack thereof, are not necessarily indicative of changes in the risk of illness associated with the product, because consumption patterns changed significantly during the study. This is demonstrated by considering the change in consumption and its effect on the implied probability of illness per serving.

**Fig. 5.**
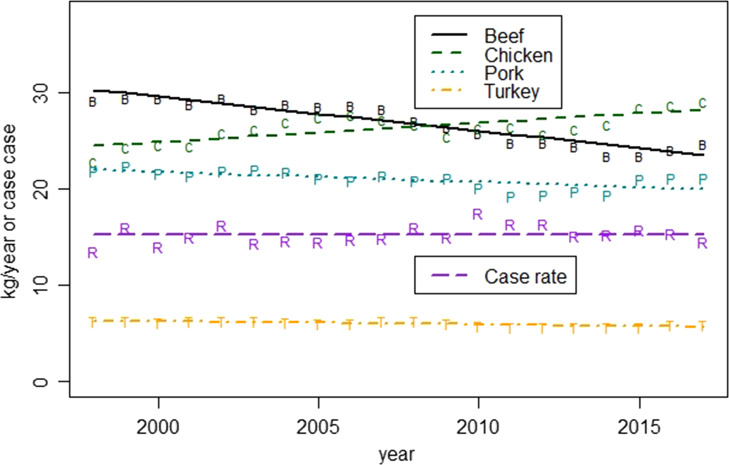
Trends in annual consumption per capita and the reported case rate per 100 000 between 1998 and 2017.

Figure 5 demonstrates that there were significant changes in the consumption for all four of the commodities of interest (*p* < 0.002). The per capita annual consumption for the commodity groups of beef, pork and turkey all declined by between 9% and 22%, while chicken was the only one of these commodities to experience an increase of 15% (Table 2). Under the assumption that the change in the proportion of outbreaks is equivalent to the change in the true attribution fraction, the change in the implied probability of illness decreases between 15% and 35% for beef, chicken and turkey, and an increase of 367% for pork (Table 2).

**Table 2.** Overall changes in consumption, proportion of outbreaks and the change in the implied probability of illness per serving for the four primary meat commodities in the United States from 1998 through 2017

Commodity	*γ*_1_	*γ*_2_	Δ*N*_*d*_	Δ*p*_*d*,*t*_	Δ*P*_*d*_(*ill*)
Beef	30.7	−0.36	−0.22	−0.49	−0.35
Chicken	24.3	0.20	0.15	−0.07	−0.19
Pork	22.1	−0.11	−0.09	3.24	3.67
Turkey	6.4	−0.03	−0.10	−0.23	−0.15

## Discussion

The available data do not support causal inferences, but the patterns observed do suggest hypotheses for further study. Of interest is the observed decrease in the annual number of outbreaks with an identified food source during the 2000–2010 period (Fig. 1). Earlier studies have identified an overall reduction in total outbreak counts for all pathogens due to changes in the surveillance system but concluded that *Salmonella* outbreaks had remained stable [25, 50]. This analysis covers a longer time period and finds a significant reduction in outbreaks with an identified food source during this period. Considering the patterns observed across all the commodities, it seems likely that much of the change is due to the reduction in the number of egg-associated outbreaks. The hypothesis is supported by the magnitude of the reduction in outbreaks associated with eggs (15 fewer outbreaks between 2000 and 2010) matched with the reduction in total outbreaks (26 fewer outbreaks between 2000 and 2010) (Table 1).

The increase in the number of outbreaks with an identified food source between 2010 and 2014 is more difficult to attribute to any one product because only the pork and seeded vegetable commodities groups were consistently increasing during this period, and neither commodity has a large number of outbreaks on par with eggs in 1999 and 2000. A reasonable hypothesis is that outbreak investigation methods may have improved, with the continued expansion and improvement of surveillance networks [51] and the widespread adoption of new technologies such as WGS into these surveillance networks [52, 53]. Also note that unidentified improvements in the sensitivity of the surveillance system would bias the estimated changes in the probability of illness associated with a commodity, similar to the changes in annual consumption.

Amongst meat and poultry commodities, the consistent and significant increase in the proportion of pork-associated outbreaks is of concern. Pork ranks as the third most frequently consumed meat commodity in the United States, yet only the chicken and the fruits–nuts commodities are responsible for a larger average proportion of outbreaks in the later years of the dataset (Table 1). This suggests that the risk of illness per serving from pork may have increased and is high relative to the other meat and poultry commodities [54]. FSIS is considering new *Salmonella* performance standards focused on raw pork products, in part because of concerns about recent *Salmonella* outbreaks linked to these products.

This analysis uses outbreak counts to estimate the fraction of outbreaks for each of the 12 commodity groups. The analysis, and its results, will likely be compared to the attribution estimates produced by IFSAC. While the 2017 estimates of the proportion of outbreaks from the compositional data models are generally similar to the 2017 attribution estimates reported by IFSAC [19] for the commodity groups that were not collapsed, direct comparisons are not appropriate because the desired inferences (i.e. trend modelling *vs.* estimation of a mean) generally require a different treatment of the available data.

While the statistics describing the annual fraction of outbreaks has the lowest signal-to-noise ratio, the possible inferences are limited by the available sample size. We would caution against inferences any more detailed than simple trends in broad commodity groups. A limitation of the study is that it does not recognise the differential risk for specific products within a commodity group (i.e. an inherent assumption of an equal probability of illness given exposure for all products within the group) [55]. For example, unpasteurised milk products [56], oysters [57], chicken livers [58] and a type of frozen breaded chicken products that appear to be cooked [59, 60] have been identified as infrequently consumed products within the dairy, fish and other seafood, and chicken commodity groups that are responsible for disproportionately large shares of outbreaks relative to their consumption.

The compositional data analysis demonstrates that significant trends exist in the proportion of outbreaks for three broad commodity groups (eggs, pork, seeded vegetables), while significant changes in the other groups were not observed. We would, however, caution against any additional interpretations beyond these results because compositional data analysis techniques can only provide information on the change in the relative values of the components [49] and the techniques cannot be used to determine which external factors led to the change. While there are more annual outbreaks of salmonellosis than any other bacterial foodborne pathogen, inferences drawn from the data for this pathogen are limited to assessing broad trends.

A limitation of the study is the inherent biases associated with all outbreak surveillance systems. Examples of factors that could affect the interpretation of the data are whether an outbreak leads to an investigation, whether specimens are collected, the types of samples and sensitivity of assays used to detect the pathogen and whether a food vehicle is identifiable for the outbreak. The degree to which these biases could have changed over the 20-year duration of the dataset is also unknown.

A further limitation of the study is that it does not recognise the differential risk for specific products within a commodity group and how these may change over time (e.g. changes in the serotype abundance and/or virulence, changes in preparation methods). This suggests that gaining insights into the effectiveness of food safety efforts undertaken by government and/or industry likely requires a more extensive effort to combine additional data throughout the food chain (e.g. data on serotypes or seasonal changes in occurrence) with data on sporadic illnesses and outbreaks. Additionally, the temporal effects observed for *Salmonella* may assist in interpreting the sparser outbreak data for other pathogens and commodities. Furthermore, the modelling effort will likely need to account for changes across the full collection of commodities to limit the effect of latent variables.

Despite the limited amount of available data and the difficulties in interpreting the results, this study demonstrates the success of efforts to reduce outbreaks associated with eggs. Furthermore, the increases observed in commodities such as pork are leading to new efforts to reduce cases of salmonellosis associated with these products, such as FSIS-proposed performance standards for raw pork products.

## Data Availability

Annual consumption and US population information is available at the USDA, Economic Research Service, Food Availability (per capita) Data System (https://www.ers.usda.gov/data-products/food-availability-per-capita-data-system/). The *Salmonella* case rate per 100 000 is provided by the Centers for Disease Control and Prevention available at https://wwwn.cdc.gov/foodnetfast/.
